# Pharmacovigilance of patients with multiple myeloma being treated with bortezomib and/or thalidomide

**DOI:** 10.1590/1414-431X20165128

**Published:** 2016-05-31

**Authors:** T.B.M. Castro, A.E. Hallack, A. Atalla, L.C. Ribeiro

**Affiliations:** 1 Programa de Pós Graduação em Saúde Brasileira, Faculdade de Medicina, Universidade Federal de Juiz de Fora, Juiz de Fora, MG, Brasil; 2 Departamento de Clínica Médica, Faculdade de Medicina, Universidade Federal de Juiz de Fora, Juiz de Fora, MG, Brasil; 3 Serviço de Hematologia e Transplante de Medula =ssea do Hospital Universitário, Faculdade de Medicina, Universidade Federal de Juiz de Fora, Juiz de Fora, MG, Brasil; 4 Departamento de Estatística, Universidade Federal de Juiz de Fora, Juiz de Fora, MG, Brasil

**Keywords:** Multiple myeloma, Side effects, Bortezomib, Thalidomide

## Abstract

In order to evaluate the main adverse effects of drug protocols using bortezomib and/or thalidomide for the treatment of multiple myeloma, we conducted a prospective study. Data were collected through interviews, clinical observation, and from hospital records. A total of 59 patients were included. There was a predominance of females, 36 (61%) *vs* 23 (39%) males, and of whites, 49 (83.1%) *vs* 10 (16.9%) blacks. Age ranged from 40 to 94 years, with a median of 65 years (SD=11.6). Regarding staging at diagnosis, 27 (45.7%) patients were in stage III-A, with 12 (20.3%) patients having serum creatinine ≥2 mg/dL. The main adverse effects in the bortezomib treatment group (n=40) were: neutropenia (42.5%), diarrhea (47.5%), and peripheral neuropathy in 60% of cases, with no difference between the *iv* (n=26) and *sc* (n=14) administration routes (P=0.343). In the group treated with thalidomide (n=19), 31.6% had neutropenia, 47.4% constipation, and 68.4% peripheral neuropathy. Neutropenia was associated with the use of alkylating agents (P=0.038). Of the 3 patients who received bortezomib in combination with thalidomide, only 1 presented peripheral neuropathy (33.3%). Peripheral neuropathy was the main adverse effect of the protocols that used bortezomib or thalidomide, with a higher risk of neutropenia in those using alkylating agents. Improving the identification of adverse effects is critical in multiple myeloma patient care, as the patient shows improvements during treatment, and requires a rational and safe use of medicines.

## Introduction

Multiple myeloma (MM) is a neoplasm with an unclear etiology, having an incidence of about 4 per 100,000, and accounting for 1% of all malignancies and 10% of hematologic malignancies (1). Treatment of the disease consists of chemotherapy, antiangiogenic agents, immunomodulators, and autologous stem cell transplants (2). Noteworthy, among the drugs that comprise the therapeutic arsenal for MM treatment are immunomodulators and proteasome inhibitors (3).

Most of the treatment protocols used in Brazil for treating MM are composed of bortezomib and/or thalidomide, combined with other drugs such as alkylating agents and corticosteroids (1). Bortezomib is a proteasome inhibitor and thalidomide is an immunomodulatory drug, and both have mechanisms of action that are not yet fully understood (4,5).

During chemotherapy, patients are highly likely to present some sort of adverse effect. Therefore, the attempt to understand the mechanisms involved in these effects, the extent of the risk, and the strategies to control them is part of an adequate patient care that the health care team should provide to patients (1,6,7). In this context, pharmacovigilance, which is an activity carried out by the professional pharmacist and is related to the detection, understanding, assessment, and prevention of adverse reactions, proves to be an important tool during chemotherapy (8). Through this method, we aimed to assess the adverse effects of drug protocols using bortezomib and/or thalidomide in patients with MM, as well as to grade and identify the main adverse reactions.

## Material and Methods

### Study design

This was a multicenter, prospective, observational study, approved by the Ethics Committee of the Hospital Universitário, Universidade Federal de Juiz de Fora. The informed consent form was signed by the researcher and patients.

The inclusion criteria for the study were: patients with MM; adults over 18 years old; elective and non-elective for bone marrow transplant and being under treatment protocols using bortezomib and/or thalidomide. There were no restrictions regarding the presence of comorbidities and use of concomitant medications.

The pharmacovigilance strategy included questionnaire-based interviews with the patient at each appointment, clinical observation, and consultation of laboratory tests and medical records.

The interviews were conducted according to the type of medication used by the patient. For example: patients that were taking bortezomib, or bortezomib and thalidomide combination were interviewed at the beginning of each cycle, and data analysis accounted for the cycle they were in; patients using only thalidomide were interviewed once a month (on the day of bisphosphonate infusion) and data analysis accounted for the number of months on medication, relating the findings to this temporal quantification.

### Toxicity monitoring

The studied adverse effects were those most frequently described in the literature and in the bortezomib and thalidomide package inserts, specifically blood and lymphatic system, gastrointestinal, and nervous system disorders. The main blood and lymphatic system disorders studied were anemia, neutropenia, and thrombocytopenia. Anemia was considered only in patients with diminished hemoglobin levels throughout the treatment. The other disorders evaluated were constipation and diarrhea, and peripheral neuropathy (PN). Blood, lymphatic system, and gastrointestinal disorders were not graded. However, PN was graded according to the National Cancer Institute (NCI) criteria, version 4.0, as shown in Table 1.

**Table:**
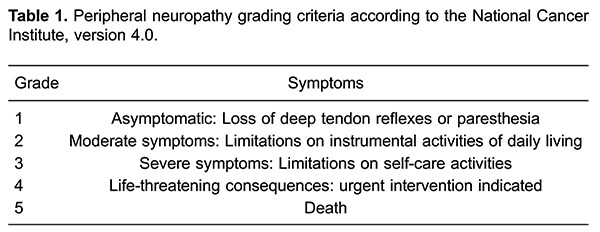

Data were collected at the University Hospital of the Federal University of Juiz de Fora, the Maria José Baeta Reis Hospital (Ascomcer), and the Radiotherapy and Nuclear Medicine Center, Ltd. (Centerq), from May 2013 to August 2015.

### Statistical analysis

For statistical analysis and database management, SPSS (Statistical Package for the Social Sciences, IBM, USA) software was used. Descriptive analysis of categorical variables was performed using frequencies, means, medians, and standard deviations. The evaluation of toxicity and tolerance was carried out using Fisher's exact test or chi-square (χ2) test for heterogeneity and for trend. A 5% significance level was adopted.

## Results

A total of 59 patients were included in the study (Table 2). As the chemotherapy protocol of 3 patients was modified during the study, 62 treatments were evaluated. Bortezomib was used in 40 patients, being administered intravenously (iv) in 26 and subcutaneously (sc) in 14. Bortezomib was combined with alkylating agents in 36 treatments. Thalidomide was used in 19 patients, 12 of them taking it in combination with alkylating agents. Protocols with bortezomib and thalidomide were used by 3 patients, and in combination with alkylating agents in 3 cases.

**Table:**
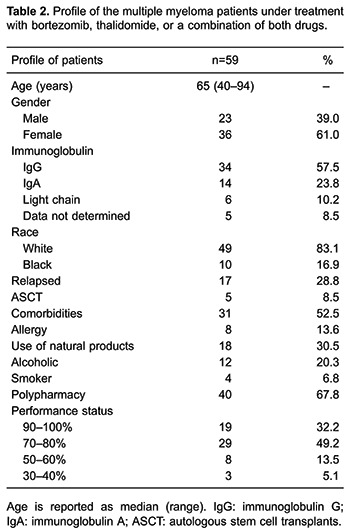

For 5 patients (8.5%), the type of immunoglobulin secreted was not determined. The most frequently found immunoglobulin was IgG, found in 34 (57.5%) patients, followed by IgA, found in 14 (23.8%), and light chain, found in 6 (10.2%).

In Table 3, we show the Durie-Salmon MM classification for the study participants, which accounts for disease stage (I, II, III) and risk (A, serum creatinine <2 mg/dL; and B, creatinine ≥2 mg/dL). Twenty-seven (45.7%) patients were in stage III-A, showing a poor prognosis and advanced illness. There were 12 (20.3%) patients with creatinine ≥2 mg/dL.

**Table:**
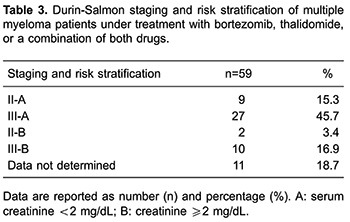

The main adverse reactions observed in patients during the study are reported in Table 4. Nervous system disorders were observed in the 3 groups of patients. PN was present in 24 (60%) patients among those treated with bortezomib protocols (without thalidomide), in 13 (68.4%) patients among those treated with thalidomide, and in 1 (33.3%) patient treated with the bortezomib + thalidomide protocol. Gastrointestinal disorders (diarrhea and/or constipation) were observed in 30 of the 40 patients treated with bortezomib protocols, with diarrhea being slightly more frequent (47.5%) than constipation (42.5%). Among the 19 patients treated with thalidomide, 13 (68.4%) had PN, 3 of them with ≥2 grade. Constipation was observed in 47.4% of patients treated with thalidomide, and was unrelated to the exposure time of the medication (P=0.370). No episodes of diarrhea were reported. Patients treated with the combination of thalidomide and bortezomib presented no gastrointestinal disorders, which may be explained by the small number of patients in this category (n=3). Table 5 shows the frequency of blood and lymphatic system disorders between patients taking or not alkylating agents, and Table 6 shows the frequency of PN between patients to whom bortezomib was administered *iv* or *sc*, excluding patients who were treated with combined bortezomib and thalidomide protocols. Two patients treated with bortezomib protocols administered intravenously showed a PN grade ≥2. In one case, there was a need for dose adjustment, and carbamazepine was indicated.

**Table:**
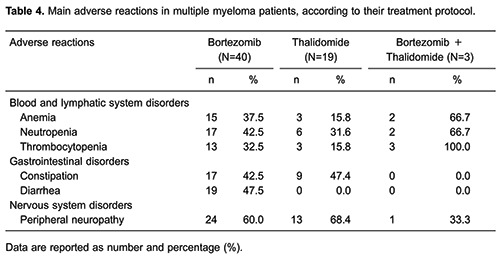

**Table:**
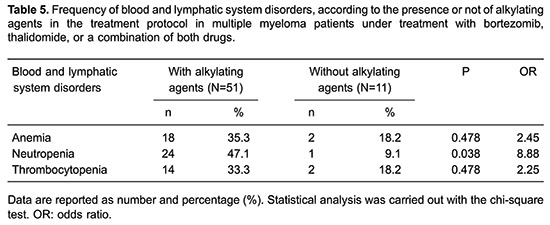

**Table:**
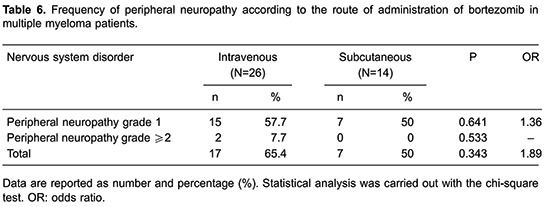

The nervous system and gastrointestinal disorders were compared between patient treatment time. The patients treated with bortezomib protocols were separated into a group with up to 4 treatment cycles *vs* a group with more than 4 treatment cycles. Meanwhile, patients submitted to thalidomide protocols were separated into a group treated for up to 4 months *vs* a group treated for more than 4 months (Table 7). The time of exposure to bortezomib and the development of nervous system disorders did not show an association (P=0.505). Patients treated with combined bortezomib and thalidomide protocols were excluded from this analysis, which was conducted independently of the bortezomib administration route.

**Table:**
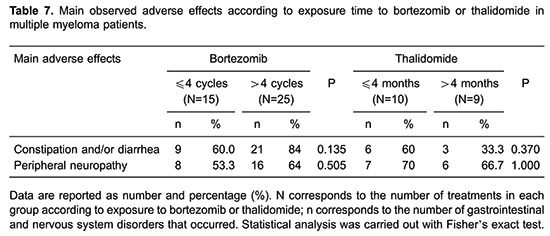

## Discussion

In this study, there was a predominance of females compared to males. These data differ from those described in the literature, where a predominance of males in MM is observed (9,10). This inconsistency may be explained by our relatively small sample size and/or specific characteristics of the study population.

The majority of the participants in our study were white, which contrasts with the literature, where a higher incidence of MM in blacks is reported (11,12). However, other studies (9,13) have reported a predominance of whites; for example, the study by Hungria et al. (13) reported a high prevalence of white/Caucasian (83.3%) in MM patients from 16 Brazilian institutions.

Patient median age of 65 years was very similar to other studies conducted in Brazil, with mean ages at diagnosis of 66 and 60.5 years (9,13). A number of studies show that the elderly are more likely to experience adverse drug reactions, which can be explained by the physiological changes that come with aging, as well as by alterations in the pharmacokinetics and pharmacodynamics of the body (14-16).

The results concerning immunoglobulins were similar to those found in a study by Kyle et al. (9). Our findings concerning disease stage were similar to a study conducted in Brazil in 2008 (13) with 1,112 patients showing that the majority of participants were already in an advanced stage of the disease at the time of diagnosis.

The most frequently found adverse reactions were blood and lymphatic system, gastrointestinal, and nervous system disorders, which are commonly observed in patients treated with chemotherapy protocols using bortezomib and/or thalidomide (17-19). Blood and lymphatic system disorders, such as leukopenia, neutropenia, and thrombocytopenia, are associated mainly with the use of alkylating agents, which act in tissues that have rapid proliferation, a high mitotic index, and a short cell cycle (17). In our study, neutropenia was significantly associated with the use of alkylating agents, however anemia and thrombocytopenia were not.

Nervous system disorders were observed in the 3 groups of patients in our study. A prospective study by Richardson et al. (19) analyzed the efficacy and safety of bortezomib for the treatment of MM, the presence of PN as a standard adverse effect, and the genetic pre-disposition of patients for the development of neuropathies. Of the 64 patients studied, 41 presented PN.

The subcutaneous *(sc)* administration of bortezomib has proven to be more efficacious compared to *iv*, in addition to reducing adverse effects, such as PN (20). Although the difference between *sc* and *iv* administration was not significant, studies show that there is a decrease of PN when bortezomib is administered *sc*, especially higher grade PN (20,21).

Gastrointestinal disorders are common side effects in patients using bortezomib (19). Similarly, in our study, gastrointestinal disorders (diarrhea and/or constipation) were observed in 75% of the patients treated with bortezomib protocols. The pathogenesis of diarrhea caused by bortezomib is not yet clear. It was believed that this proteasome inhibitor did not cause mucosal damage, but a recent report has described mucositis in the colon of patients under bortezomib treatment (22).

Mileshkin et al. (23) conducted a study to evaluate PN in MM patients being treated with thalidomide. Among 75 patients, 41% developed PN and 15% discontinued treatment due to this toxicity. The relationship between the exposure time to thalidomide and PN is still not fully clarified (23). Our findings showed that the time of exposure to thalidomide and PN was not significantly associated. Constipation was a common adverse reaction in patients treated with thalidomide, which is in accordance to other studies. It has been hypothesized that this gastrointestinal disorder is a side effect of thalidomide action on the nerve endings of the intestine, as occurs with other neurotoxic agents such as vincristine (18).

Our overall findings were similar to those from Brazilian and international literature (13,19,23), showing a high frequency of side effects. Although we have not assessed the impact on quality of life of our patients, we infer that such events have a negative impact, leading us to believe that the improvement in the identification of adverse events is extremely important for the care of the MM patient. Since the majority of the adverse drug reactions are nonspecific, they may very often be confused with symptoms of the disease or from other causes. Studies on pharmacovigilance should be encouraged in order to achieve a better understanding of these adverse effects, which will allow for a greater adherence to treatment and consequently improve prognosis and quality of life for these patients.
